# Impact of different cover letter content and incentives on non-response bias in a sample of Veterans applying for Department of Veterans Affairs disability benefits: a randomized, 3X2X2 factorial trial

**DOI:** 10.1186/s12874-022-01531-x

**Published:** 2022-03-06

**Authors:** Maureen Murdoch, Barbara A. Clothier, Timothy J. Beebe, Ann K. Bangerter, Siamak Noorbaloochi

**Affiliations:** 1 Section of General Internal Medicine, Minneapolis VA Health Care System, One Veterans Drive (111-0), Minneapolis, MN 55417 USA; 2 Center for Care Delivery and Outcomes Research, Minneapolis VA Health Care System, One Veterans Drive (152), Minneapolis, MN 55417 USA; 3 Department of Internal Medicine, University of Minnesota Medical School, 420 Delaware St SE, Minneapolis, MN 55455 USA; 4 Division of Health Policy and Management, University of Minnesota School of Public Health, 420 Delaware St SE, Minneapolis, MN 55455 USA

**Keywords:** Non-response Bias, Randomized trial, Leverage salience theory, Factorial design, Mailed survey, Combat, Military sexual trauma, Sexual assault

## Abstract

**Background:**

Non-random non-response bias in surveys requires time-consuming, complicated, post-survey analyses. Our goal was to see if modifying cover letter information would prevent non-random non-response bias altogether. Our secondary goal tested whether larger incentives would reduce non-response bias.

**Methods:**

A mailed, survey of 480 male and 480 female, nationally representative, Operations Enduring Freedom, Iraqi Freedom, or New Dawn (OEF/OIF/OND) Veterans applying for Department of Veterans Affairs (VA) disability benefits for posttraumatic stress disorder (PTSD). Cover letters conveyed different information about the survey’s topics (combat, unwanted sexual attention, or lifetime and military experiences), how Veterans’ names had been selected (list of OEF/OIF/OND Veterans or list of Veterans applying for disability benefits), and what incentive Veterans would receive ($20 or $40). The main outcome, non-response bias, measured differences between survey respondents’ and sampling frame’s characteristics on 8 administrative variables, including Veterans’ receipt of VA disability benefits and exposure to combat or military sexual trauma. Analysis was intention to treat. We used ANOVA for factorial block-design, logistic, mixed-models to assess bias and multiple imputation and expectation-maximization algorithms to assess potential missing mechanisms (missing completely at random, missing at random, or not random) of two self-reported variables: combat and military sexual assault.

**Results:**

Regardless of intervention, men with any VA disability benefits, women with PTSD disability benefits, and women with combat exposure were over-represented among respondents. Interventions explained 0.0 to 31.2% of men’s variance and 0.6 to 30.5% of women’s variance in combat non-response bias and 10.2 to 43.0% of men’s variance and 0.4 to 31.9% of women’s variance in military sexual trauma non-response bias. Non-random assumptions showed that men’s self-reported combat exposure was overestimated by 19.0 to 28.8 percentage points and their self-reported military sexual assault exposure was underestimated by 14.2 to 28.4 percentage points compared to random missingness assumptions. Women’s self-reported combat exposure was overestimated by 8.6 to 10.6 percentage points and military sexual assault exposure, by 1.2 to 6.9 percentage points.

**Conclusions:**

Our interventions reduced bias in some characteristics, leaving others unaffected or exacerbated. Regardless of topic, researchers are urged to present estimates that include all three assumptions of missingness.

**Supplementary Information:**

The online version contains supplementary material available at 10.1186/s12874-022-01531-x.

## Background

Not everyone invited to participate in mailed surveys will do so, particularly when the survey’s content is sensitive [1]. When those who opt out of a survey differ systematically from those who participate, bias may enter one’s dataset [2]. Missing survey units are typically categorized as missing completely at random, missing at random, or not at random [3]. Only the first category can be ignored in analysis. For the remaining two, researchers must rely on computationally intensive, post-experimental balancing procedures to correct any potential biases. For missing at random situations, one might consider weighting, matching, stratification, multiple imputation, or propensity score adjustment to address the mechanism of missingness [4, 5]. When missingness is non-random or informative, analysis requires considerably more complex modeling procedures and expert statistical input [6]. There are few off-the-shelf statistical packages to address non-random missingness, in part because the mechanisms and patterns of missingness can vary substantially from project to project. Thus, users must often write their own computer programs for model estimation (see [7]). Even relatively simple models may become computationally prohibitive very quickly. We previously showed that male Gulf War I Veterans applying for posttraumatic disability benefits were substantially less likely than other men to return a survey asking about military sexual assault if they were sexual assault survivors [8]. Accounting for this non-ignorable non-response bias using Bayesian [4], maximum likelihood [9], and expectation-maximization [10, 11] techniques required intensive computer time and resources. Besides being time- and resource-intensive, analytical remedies for missingness must be applied after data collection ends, when the underlying biases can no longer be rectified.

According to Leverage-Salience Theory [12], many considerations prompt or dissuade people to take part in mailed surveys, with different people potentially viewing any given factor quite differently. How individuals judge a particular survey aspect—either positively or negatively—and how much weight or importance they place on that aspect are known as “leverages.” Interest in the survey’s topic and monetary incentives are two common leverages that typically encourage people to complete and return surveys [13]. Sensitive or high-threat questions, such as those asking about sexual behavior or personal finances, are examples of negative leverages that may discourage survey participation [1, 14]. Highlighting or emphasizing selected information about a research effort influences which leverages participants attend to. This is the “salience” part of the theory, since to highlight information is to make it more activating or salient to participants. Several avenues can be exploited in mailed surveys to highlight or activate selected leverages [13], including pre-notification letters, the questionnaire’s design, or, as in the present study’s focus, the cover letter.

Consistent with Leverage-Salience Theory, we previously showed that specifically mentioning a survey’s combat content in a cover letter resulted in over-representation from male combat Veterans, even when—perhaps especially when—other factors thought to suppress response rates, such as lower incentives and less privacy, were implemented [15]. In other work, we observed no difference in disability benefit status between Veteran respondents and non-respondents when we told survey recipients their name had been “randomly selected from an electronic database of Veterans” [16] but an almost 3-fold over-representation of disability benefit recipients when we informed survey recipients their name had been selected from a “list of Veterans filing disability claims” [15]. In the present study, our goal was to see if modifying key cover letter information might prevent non-random non-response bias and its attendant need for time-consuming, complicated, post-survey modeling procedures. The study extends our previous investigations into non-response bias in studies involving Veterans applying for disability benefits.

One cannot measure non-response bias without knowing something about the population of interest. Unfortunately, in many studies, population-level information is obtained from other sources, such as the United States Census, where differences in sampling approaches, question asking, and timeframe may introduce methodologically artifactual estimates of bias [5]. When sampling frame data are available, information is often limited to just a few sociodemographic characteristics, which, in turn, are only marginally related to study outcomes [5]. In the present study we take advantage of the Department of Veterans Affairs’ (VA) data warehouse to build a rich sampling frame of characteristics that we hypothesized would be related to survey non-response and to the receipt of VA disability benefits. Known predictors of receiving VA disability benefits for posttraumatic stress disorder (PTSD) include older age, male gender, and combat exposure [17, 18]; negative predictors include female sex, non-white race, and history of military sexual assault [16, 18]. Greater medical and psychiatric comorbidity are associated with receipt of any VA disability benefits [19]. We hypothesized that specifically telling Veterans that the survey asked about combat would trigger over representation of combat Veterans, that specifically mentioning military sexual assault would result in under-representation from male sexual assault survivors and possibly female sexual assault survivors, and that providing a more generic description of the survey’s content would generate the most representative respondent pool. We also hypothesized that telling survey recipients their name had been selected from a list of Veterans applying for VA disability benefits would result in over-participation by disability benefit recipients (and thus over-participation by Veterans with more medical and psychiatric comorbidities), while giving a less specific accounting of where their name came from would result in more representative participation.

Our secondary goal was to examine the effects of different incentives on non-response bias. Incentives have consistently been shown to increase survey response rates, e.g., [20] but the impact on non-response bias is less clear, e.g., [21–24]. In prior work with male disability applicants, we showed that larger incentives tended to attract younger, healthier, working men compared to smaller incentives [15]. We anticipated that a larger incentive in the present study would also reduce non-response bias related to age, health, and disability status.

## Methods

### Study design and human studies oversight

The study is a gender-blocked, randomized, 3X2X2 factorial comparison trial. The Minneapolis VA Health Care System’s Internal Review Board for Human Studies reviewed and approved the study protocol (#4495-B). All analyses were pre-planned. Data were collected between February and August 2016.

### Participants

Participants were Veterans who had served during Operations Enduring Freedom, Iraqi Freedom, and New Dawn (OEF/OIF/OND) and had a pending VA disability claim for PTSD. From a sampling frame of 14,630 men and 2945 women, we randomly selected, without replacement, 480 men and 480 women to receive mailed surveys.

Selected Veterans had a median age of 33.0 years (interquartile range = 12, mean = 35.2, SD = 8.9, range 19–67) and 78.0% had received combat pay while on active duty. In terms of health, 6.7% had been diagnosed with bipolar disorder, schizophrenia, or schizoaffective disorder; 10.8% had Charlson Comorbidity Index [25] scores greater than zero.

### Protocol

Veterans received pre-notification letters 1 week before we mailed a cover letter and 22-page questionnaire to their homes. The questionnaire asked about PTSD and depression symptoms; functioning; pain; substance use; and traumatic exposures in the military, including combat and military sexual assault. Of these items, only self-reported combat and military sexual assault are considered here.

Cover letters were mailed with the questionnaires. All cover letters used the same language to describe the risks and benefits of participating in the research and to emphasize that participation was voluntary. At two-week intervals, non-respondents received postcard reminders; a follow-up mailing of the questionnaire; and a third, final mailing of the questionnaire via United Parcel Service’s 3-day delivery service. Veterans signified their consent to participate by returning a completed survey. Except for specific cover letter content described in “Study Arms” below, all other aspects of the survey were the same across groups, including the pre-notification letters, reminder postcards, and questionnaires.

### Study arms

We used the individual cover letters to deliver the stimulus. As shown in Table 1, the first study factor varied the information survey recipients received in the cover letter about the survey’s topics. Veterans were told that the survey would ask about “combat,” about “unwanted sexual attention while in the military,” or about “lifetime and military experiences that can affect well-being.” The second factor varied the cover letter’s information about how Veterans’ names were obtained: either from a “Department of Veterans Affairs list of Veterans who served during OEF/OIF/OND” or from a “Department of Veterans Affairs list of Veterans who filed a disability claim.” The third factor examined the effect of different incentives: $20 or $40. Because of local policies, incentives were paid only to Veterans who returned a completed survey. Veterans were told which incentive they would receive in the cover letter.

**Table 1:** Study Factors and Allocation of Participants

		Factor 1: What Veterans were told about the survey’s content					
		Survey Asks about Combat		Survey asks about unwanted sexual attention		Survey asks about lifetime and military experiences	
		Factor 3: Honoraria		Factor 3: Honoraria		Factor 3: Honoraria	
		$20	$40	$20	$40	$20	$40
Factor 2: What Veterans were told about how their name was obtained for the study	Name obtained from list of OEF/OIF/OND Veterans	*n* = 80 40 men, 40 women	*n* = 80 40 men, 40 women	*n* = 80 40 men, 40 women	*n* = 80 40 men, 40 women	*n* = 80 40 men, 40 women	*n* = 80 40 men, 40 women
	Name obtained from a list of Veterans applying for disability benefits	*n* = 80 40 men, 40 women	*n* = 80 40 men, 40 women	*n* = 80 40 men, 40 women	*n* = 80 40 men, 40 women	*n* = 80 40 men, 40 women	*n* = 80 40 men, 40 women

*OEF/OIF/OND* Operation Enduring Freedom, Operation Iraqi Freedom, Operation New Dawn

After blocking on gender, we randomly divided the 480 men into 12 equal-sized groups of 40 individuals. Each of the 12 possible cover letter iterations (i.e., what recipients were told about the survey’s topic, how their name was obtained, and what incentive they would receive) was then randomly assigned to one of the 12 groups of men. This same procedure was repeated for the 480 women (see [26]). Randomizations were accomplished using a computer-generated program, overseen by BAC. The remaining co-authors were unaware of allocation until after it was completed.

### Outcomes

#### Main outcome

The main outcome was non-response bias on each of our 8 pre-specified non-response correlates (see “Measures,” below).

#### Secondary outcomes

Secondary outcomes included unit (survey) response, the percent of non-response variance in our 8 correlates that was explained by the different cover letter and incentive iterations, and the impact of different missingness assumptions on the estimated prevalence of combat and military sexual assault exposures.

### Measures

#### Non-response correlates

As mentioned previously, the characteristics we anticipated would be related to survey non-response and to the receipt/non-receipt of VA disability benefits included age, race/ethnicity, combat exposure, history of military sexual assault, and greater medical or psychiatric comorbidity. Veterans’ VA disability benefit status was of itself thought to predict survey non-response. Indicators for all these variables were available for the entire sampling frame through the VA’s Corporate Data Warehouse. Age was dichotomized as < or ≥ to 30 years. We used the Race/ethnicity data fields from the Veterans Benefits Administration, which categorizes Veterans into 7 mutually exclusive categories including, “Asian or Pacific Islander,” “Black or African American,” “Hispanic ethnicity,” “Other,” “Unknown,” or “White.” For descriptive analyses we combined the “Other” and “Unknown” categories. When calculating bias, we dichotomized race as “Non-White” and “White.” We used combat flags, medical diagnoses, special issue codes, and Veterans’ responses to the VA’s military sexual trauma screener to categorize them as having combat or military sexual trauma exposure. “Military sexual trauma” encompasses sexual assault and severe, pervasive physical sexual harassment while in the Armed Forces. Results were dichotomized as “exposed” or “not exposed.” We used inpatient and outpatient VAICD-9-CM and ICD-10-CM codes for bipolar disorder, schizoaffective disorder, or schizophrenia to determine Veterans’ serious mental illness status, dichotomized as “present” or “not present,” in the 180 days prior to survey. We likewise used inpatient and outpatient VA ICD-9-CM and ICD-10-CM codes to calculate Charlson Comorbidity Index [25] scores for each Veteran. We dichotomized results as 0 versus ≥1. Veterans with no VA health care utilization received Charlson Comorbidity Scores of 0 (*n* = 48).

Veterans’ disability claims were pending at the time of survey; therefore, their benefit status for any disorder or for PTSD specifically was ascertained approximately 7 months after the survey was fielded. We dichotomized results as “receiving disability benefits” or “not receiving benefits.”

#### Self-reported exposures

Self-reported combat exposure and military sexual assault were available for respondents’ only. We used the Combat Experiences subscale of the Deployment Risk and Resilience Inventory-2 [27] to assess combat exposures and a 5-item adaptation of the Sexual Harassment Inventory’s [28] Criminal Sexual Misconduct subscale to assess military sexual assault. Note that military sexual trauma, obtained from VA administrative data, is not equivalent to self-reported military sexual assault, as the former term also encompasses severe, physical sexual harassment. Self-reported outcomes for combat and for military sexual assault were dichotomized as “any” versus “none.”

### Analysis

Analysis was intention to treat. Results are reported separately by gender to account for our sampling strategy. The primary outcome, non-response bias, was calculated as the difference in the percentage of people with each non-response correlate in the respondent sample minus the percentage of people with the same characteristic in the sampling frame. Negative numbers indicate under representation of that characteristic in the respondent pool, and positive numbers indicate over representation. Zero indicates no bias.

We used the American Association for Public Opinion Research’s [29] response rate definition #1 to calculate survey response rate. Specifically, response rate was calculated as the number of returned surveys in each condition divided by the number of Veterans assigned to that condition. We dichotomized unit response as “survey returned” versus “not returned.”

We used ANOVA to see if the mean bias differed statistically significantly across the 3 main interventions. The associated degrees of freedom for non-significant interaction terms were added to the final model’s error term. We took advantage of the ability to partition the sum of squares in ANOVA to determine the percent of non-response variance in our 8 correlates explained by the 3 study-arm manipulations. The Intervention (study-arm manipulations) Sum of Squares (SSI) divided by the Total Sum of Squares (SST), or η^2^, multiplied by 100% represents the variance explained by each of the study arm manipulations.

Following a similar approach to Murdoch, et al. [8], we used the survey’s two self-report variables (combat exposure and military sexual assault) to identify the study-arm manipulations’ impact on missing mechanisms (i.e., missing completely at random, missing at random, or non-random). Numerically close estimates and small variances across the 3 resulting estimates would support random missingness. We used observed values to estimate prevalence under missing completely at random assumptions, 25 copies of imputed values to estimate the prevalence of military sexual assault and combat exposure under the missing at random assumption [30] and Ibrahim and Lipsitz’s [10, 11] expectation–maximization algorithm to estimate prevalences under a not random assumption. Ibrahim and Lipsitz’s method assumes that missingness in the outcome variable is related to recorded covariates that are assumed or known to be associated with the outcome. This information is then used to estimate the joint probability of being a non-respondent and of having the outcome of interest.

We used SPSS version 19.0, SAS version 9.4, and R version 4.0.2 for analyses and graphics.

### Power

For each of the 3 study manipulations, the study had 80% power a priori to detect a bias between respondents and the underlying sampling frame ranging from 5 to 12 percentage points, depending on the population’s prevalence. For conditions expected to be very common (e.g., 90% combat exposure or 90% service connection in men [8, 18]) or uncommon (e.g., 2% military sexual trauma in men [31]), the smallest detectable bias was estimated to be 5 percentage points. For intermediate prevalences (e.g., 30% combat exposure in women or 50% service connection in women [18]) the smallest detectable bias was 12 percentage points. Biases in this range approximate Cohen’s *h* = 0.25, generally considered a small to moderate effect [32]. We assumed a response rate of 50% and two-tailed alpha = 0.05.

## Results

### Response rate

Response rates by each study-arm manipulation and gender are shown in Table 2. A total of 410 Veterans (42.7%) returned completed surveys, with an overall response rate of 41.5% for men and 44.0% for women. As can be seen from Table 2, emphasizing different survey topics in the cover letter, such as combat or unwanted sexual attention, did not significantly influence either gender’s net survey response. Men, but not women, were more likely to return a survey if told their name had been obtained from a list of Veterans applying for VA disability benefits compared to a list of OEF/OIF/OND Veterans. Both men and women were statistically significantly more likely to return a survey if they were promised a $40 post-paid incentive compared to $20. Supplementary Table 1 (Additional File) shows response rates for each factorial combination by gender. Men’s survey response rates ranged from 22.5 to 60%, depending on their factorial combination, and women’s, from 32.5 to 60%. The lowest response rates for both genders were obtained from those promised a $20 post-paid incentive, told that the survey asked about lifetime and military experiences, and informed that their name had been obtained from a list of OEF/OIF/OND Veterans. Omnibus χ^2^ tests indicated that men’s response rates differed significantly across the different factorial combinations (*p* = 0.03), but women’s response rates did not (*p* = 0.88).

**Table 2:** Survey response (n) and response rate (%) by gender and study-arm manipulation

Gender	Overall	What Veterans were told about content			How name was obtained		Honoraria	
		Combat	Unwanted Sexual Attention	Lifetime/Military Experiences	List of OEF/OIF/OND Veterans	List of Veterans Applying for Disability Benefits	$20	$40
Men	199 (41.5%)	70 (43.8%)	69 (43.1%)	60 (37.5%)	**88 (36.7%)**	111 (46.3%)*	**86 (35.8%)**	113 (47.1%)*
Women	211 (44.0%)	64 (40.0%)	75 (46.9%)	72 (45.0%)	102 (42.5%)	109 (45.4%)	**94 (39.2%)**	117 (48.8%)*

*OEF/OIF/OND* Operation Enduring Freedom, Operation Iraqi Freedom, Operation New Dawn

**Bold face font** signifies a statistically significant different response rate within study-arm manipulation within gender

*p < 0.05

### Non-response bias

Table 3 shows the sampling frame’s characteristics stratified by gender and survey response status. As can be seen, on net, male non-respondents were statistically significantly more likely to be under age 30 and less likely to have any VA disability benefits compared to survey respondents. Female non-respondents were statistically significantly less likely to have a serious mental illness and less likely to have VA disability benefits for PTSD compared to respondents. There was trend for women without combat exposure to be non-respondents (*p* = 0.06).

**Table 3:** Sample Characteristics by Gender and Response Status

Characteristic as obtained from VA databases	Men			Women		
	Overall	Respondent?		Overall	Respondent?	
		No	Yes		No	Yes
	*N* = 480	*n* = 281	*n* = 199	*N* = 480	*n* = 269	*n* = 211
Age < 30	30.4	35.2***	**23.6**	32.3	34.2	29.9
Race						
White	58.1	58.0	58.3	40.6	37.2	45.0
Black	19.6	19.6	19.6	36.5	37.9	34.6
Hispanic	13.3	11.7	15.6	10.2	11.5	8.5
Asian	6.5	7.1	5.5	6.3	6.0	6.6
Other/Unknown	2.5	3.6	1.0	6.5	7.4	5.2
Combat exposure	67.1	67.3	66.8	41.3	37.6	46.0
Military sexual trauma exposure	2.1	2.1	2.0	45.6	46.5	44.6
Serious mental illness	5.2	4.1	6.7	8.2	5.6*	**11.4**
Charlson Comorbidity Index > 0	11.7	10.7	13.1	10.0	9.3	10.9
VA disability benefits for PTSD	56.3	54.8	58.3	45.2	40.5*	**51.2**
Any VA disability benefits	81.9	78.3*	**86.9**	77.3	75.5	79.6

Results reported as Column Percentages (%). *VA* Department of Veterans Affairs, *PTSD* Posttraumatic

stress disorder. **Bold face font** signifies a statistically significant difference between respondents and non-

respondents within gender

*p ≤ 0.05, ** *p* ≤ 0.01, ***p ≤ 0.001

**Fig. 1:**
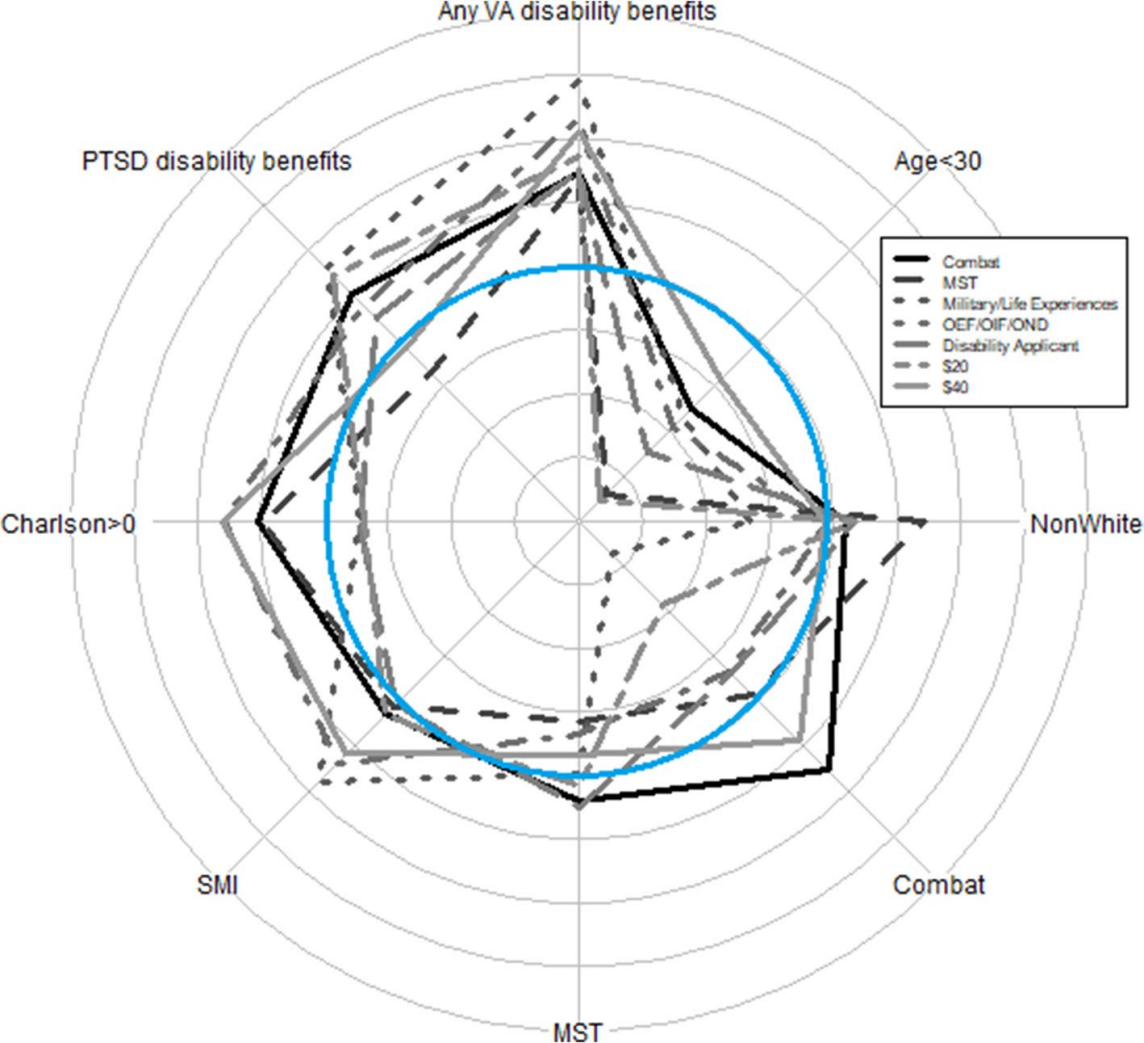
Bias in the 8 non-response correlates in men by the 7 study-arm manipulations. Grid lines range from − 12 (the center point) to + 12 percentage points. Negative numbers indicate under-representation of the characteristic in respondents compared to the sample, and positive numbers, over-representation. The blue circle represents 0 or no bias. MST = military sexual trauma. OEF/OIF/OND = Operation Enduring Freedom, Operation Iraqi Freedom, Operation New Dawn. SMI = Serious mental illness diagnosis

**Fig. 2:**
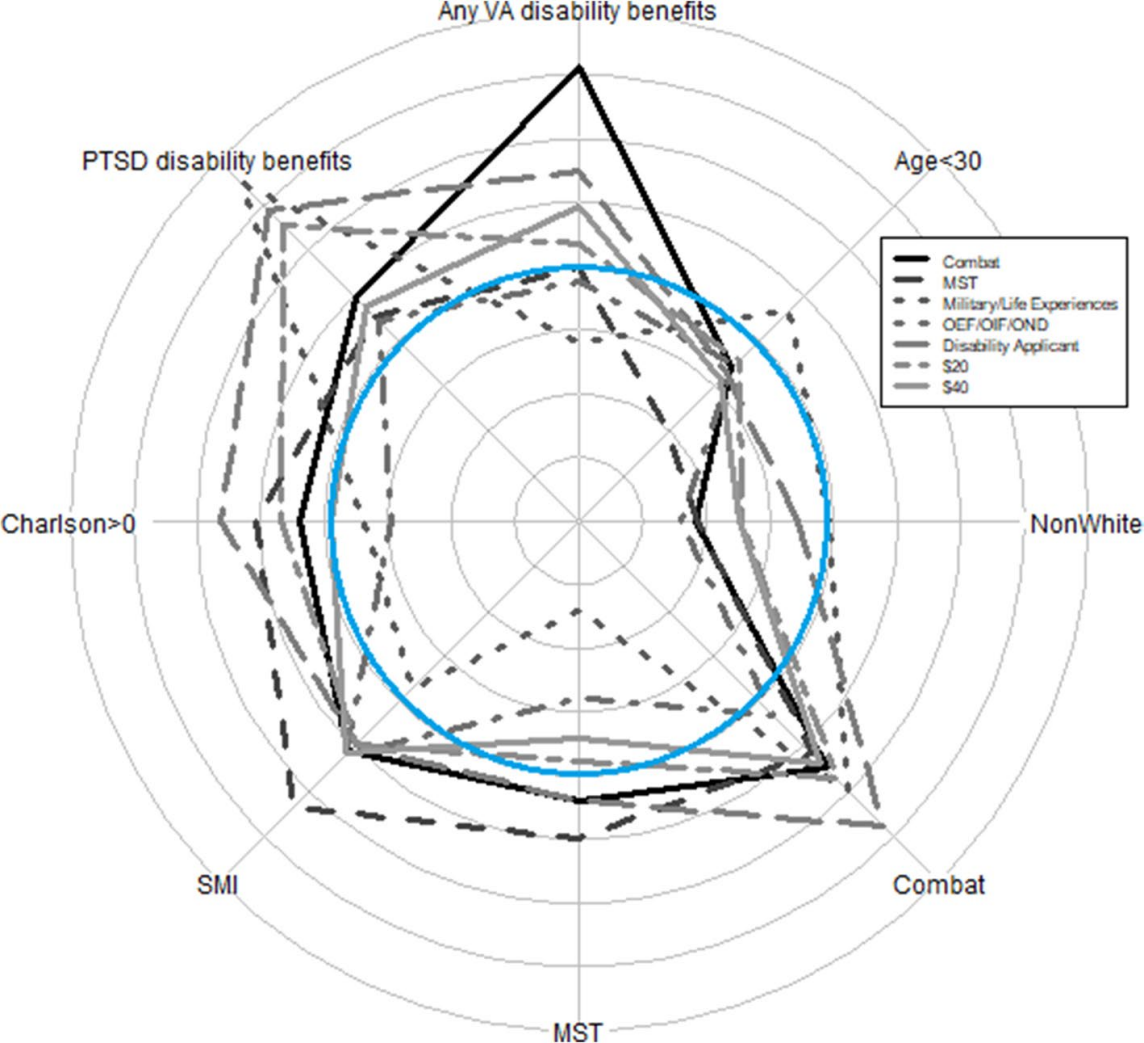
Bias in the 8 non-response correlates in women by the 7 study-arm manipulations. Grid lines range from − 12 (the center point) to + 12 percentage points. Negative numbers indicate under-representation of the characteristic in respondents compared to the sample, and positive numbers, over-representation. The blue circle represents 0 or no bias. MST = military sexual trauma. OEF/OIF/OND = Operation Enduring Freedom, Operation Iraqi Freedom, Operation New Dawn. SMI = Serious mental illness diagnosis

Different interventions could, of course, cancel one another out, resulting in a null effect on net non-response bias. Figures 1 and 2, therefore, show the degree to which men and women with the 8 study characteristics were over- or under-represented within each study-arm manipulation. Supplementary Tables 2a and 2b **(**Additional file**)** provide the same information in tabular form. As Fig. 1 shows, across all the interventions, men under age 30 were consistently under-represented among respondents, while men with serious mental illness and men with any VA disability benefits were consistently over-represented. Men with combat exposure were most over-represented in the group told the survey asked about combat, and men with military sexual trauma were most under-represented in the group told the survey asked about unwanted sexual attention. Although these latter two effects were in the direction expected, neither was statistically significant.

Contrary to our expectations, men with serious mental illness and higher Charlson scores were over-represented among those told their name had been obtained from a list of OEF/OIF/OND Veterans compared to those told their name came from a list of Veterans applying for disability benefit; again, these differences were not statistically significant. Compared to the $20 post-paid incentive, offering $40 was not associated with statistically significant differences in bias across any of the 8 administrative variables, though men under age 30 were least under-represented in that study arm.

As Fig. 2 shows, in contrast to the men, women with combat exposure were consistently over-represented among survey respondents, regardless of study-arm manipulation. Non-white women were consistently under-represented in all the study arms, and women receiving VA disability benefits for PTSD were consistently over-represented. Women with military sexual trauma were under-represented among those told the survey asked about lifetime and military experiences compared to those told the survey asked about combat or about unwanted sexual attention, but the difference was not statistically significant (*p* = 0.22). Different from the men but as we had hypothesized, women with higher Charlson scores were statistically significantly over-represented in the group told their name had been obtained from a list of Veterans applying for disability benefits compared to those told their name came from a list of OEF/OIF/OND Veterans. However, opposite expectations, women with serious mental illness were similarly over-represented in both groups. As with the men, offering $40 was not associated with any statistically significant differences in bias compared to offering $20. Although we had no specific hypothesis tied to it, bias in the percentage of women receiving any VA disability benefits was statistically significantly different across the three groups receiving different information about the survey’s topic (*p* = 0.04).

### Variance in non-response bias explained by the study-arm manipulations

Table 4 shows how much of the variance in non-response bias was explained by each of the three study-arm manipulations. This ranged from negligible to substantial, depending on the characteristic and intervention. Different descriptions of the cover letter content explained 31.2% of the variance in men’s combat non-response bias and 25.6% of the variance in men’s military sexual trauma non-response bias; in women, the different descriptions explained just 2.2% of the variance in women’s combat non-response but 31.9% of the variance in military sexual trauma non-response bias. Almost a third of the variance in non-white women’s under-representation was explained by how we said their name was obtained; this study-arm manipulation also explained more than 50% of the non-response bias in women with Charlson scores > 0. Differences in post-paid incentives had mostly negligible impact on women’s bias in any of the 8 administrative variables but explained 36.6% of the variance in men’s non-response bias by age, 18.1% of the variance in men’s combat exposure bias, and 19.1% of the bias in men’s Charlson scores > 0.

**Table 4:** Variance in bias (η^2^) explained by each study-arm manipulation, stratified by gender

Characteristic as obtained from VA databases	What Veterans were told about content		How name was obtained		Honoraria	
	Men	Women	Men	Women	Men	Women
Age < 30	14.5%	24.2%	1.4%	0.1%	36.6%	0.9%
Non-white race	23.1%	38.9%*	0.7%	34.2%*	1.2%	0.0%
Combat exposure	31.2%	2.2%	0.0%	30.5%	18.1%	0.6%
Military sexual trauma exposure	25.6%	31.9%	43.0%	8.3%	10.2%	0.4%
Serious mental illness	12.4%	34.8%	9.1%	0.4%	4.1%	0.4%
Charlson Comorbidity Index > 0	8.5%	14.4%	17.4%	53.8%*	19.1%	4.3%
Any VA disability benefits	9.0%	52.0%*	3.1%	11.8%	0.7%	1.4%
VA disability benefits for PTSD	11.2%	17.4%	0.2%	15.5%	7.2%	8.6%

*VA* Department of Veterans Affairs, *PTSD* posttraumatic stress disorder

*Bold face font indicates there was a net non-response bias for that characteristic and study-arm manipulation at *p* < 0.05

### Impact of different missing mechanisms on estimates of combat and military sexual assault

Tables 5 and 6 show how estimates of men and women’s combat and military sexual assault exposure based on self-report changed across the 7 study-arm manipulations, depending on the missingness assumption used. Exposures based on VA administrative data are listed for reference. As Table 5 shows, when assuming random missingness compared to non-random missingness, men’s combat exposure was overestimated by 19.0 to 28.8 percentage points, and their military sexual assault exposure was underestimated by 14.2 to 28.4 percentage points. For women (Table 6), combat exposure was overestimated by 8.6 to 10.6 percentage points when assuming random missingess compared to non-random missingness, but their military sexual assault exposure was overestimated by only 1.2 to 6.9 percentage points when comparing random to non-random missingness.

**Table 5:** Percentage of Men with Self-Reported Combat and Military Sexual Assault Experiences by Different Missingness Mechanisms and Study-Arm Manipulation

Characteristic	Overall *N* = 480	What Veterans were told about content			How name was obtained		Honoraria		Range across manipulations
		Combat *n* = 160	Unwanted Sexual Attention *n* = 160	Lifetime/military experiences *n* = 160	List of OEF/OIF/OND Veterans *n* = 240	List of Veterans Applying for disability benefits *n* = 240	$20 *n* = 240	$40 *n* = 240	
**Self-reported combat if**									
Missing completely at random		89.3%	93.0%	91.1%	95.5%	83.8%	90.1%	92.3%	9.2
Missing at random		85.3%	91.3%	88.0%	94.2%	82.2%	86.6%	89.9%	12.0
Not missing at random		66.8%	69.8%	64.7%	76.5%	56.2%	61.3%	72.5%	20.3
Variance across the 3 missingness assumptions		0.0144	0.0167	0.0208	0.0113	0.0240	0.0247	0.0117	0.0134
Range across the 3 assumptions		22.5	23.2	26.4	19.0	27.6	28.8	19.8	9.4
Combat exposure per VA administrative data	67.1%	65.0%	73.1%	63.1%	65.8%	68.3%	65.0%	69.2%	10.0
**Self-reported military sexual assault if**									
Missing completely at random		5.2%	5.6%	9.8%	5.6%	7.7%	10.4%	4.1%	6.3
Missing at random		5.6%	6.1%	9.5%	6.0%	8.1%	9.7%	4.4%	5.3
Not missing at random		25.6%	21.9%	35.6%	27.0%	27.7%	38.8%	18.3%	20.5
Variance across the 3 missingness assumptions		0.0136	0.0086	0.0224	0.0150	0.0131	0.0276	0.0066	0.021
Range across the 3 assumptions		20.4	16.3	25.8	21.4	20.0	28.4	14.2	14.2
Military sexual trauma exposure per VA administrative data^a^	2.1%	3.1%	1.3%	1.9%	2.1%	2.1%	1.7%	2.5%	1.4

*VA* Department of Veterans Affairs. ^a^VA administrative data assesses military sexual assault plus severe sexual harassment

**Table 6:** Percentage of Women with Self-Reported Combat and Military Sexual Assault Experiences by Different Missingness Mechanisms and Study-Arm Manipulation

Characteristic	Overall *N* = 480	What Veterans were told about content			How name was obtained		Honoraria		Range across manipulations
		Combat *n* = 160	Unwanted Sexual Attention *n* = 160	Lifetime/military experiences *n* = 160	List of OEF/OIF/OND Veterans *n* = 240	List of Veterans Applying for disability benefits *n* = 240	$20 *n* = 240	$40 *n* = 240	
**Self-reported combat if**									
Missing completely at random		64.2%	63.5%	73.5%	66.8%	67.7%	67.0%	67.4%	10.0
Missing at random		62.3%	60.6%	71.4%	65.8%	63.7%	63.9%	65.6%	10.8
Not missing at random		55.6%	53.2%	63.7%	56.8%	58.3%	56.4%	58.7%	10.5
Variance across the 3 missingness assumptions		0.00197	0.00282	0.00266	0.00303	0.00223	0.00297	0.00211	0.00106
Range across the 3 assumptions		8.6	10.3	9.8	10.0	9.4	10.6	8.7	2.0
Combat exposure per VA administrative data (%)	41.3%	40.6%	37.5%	45.6%	38.8%	43.8%	43.3%	39.2%	6.3
**Self-reported military sexual assault if:**									
Missing completely at random		50.1%	64.3%	52.5%	54.3%	57.2%	61.2%	50.2%	14.2
Missing at random		51.0%	66.5%	51.6%	54.8%	57.9%	60.8%	51.9%	15.5
Not missing at random		49.8%	59.6%	47.5%	50.7%	54.0%	57.3%	47.3%	12.3
Variance across the 3 missingness assumptions		0.00004	0.00124	0.00071	0.00050	0.00043	0.00046	0.00054	0.0012
Range across the 3 assumptions		1.2	6.9	5.0	4.1	3.9	3.9	4.6	5.7
Military sexual trauma exposure per VA administrative data^a^	45.6%	43.8%	45.0%	48.1%	45.5%	45.8%	45.4%	45.8%	4.3

*VA* Department of Veterans Affairs. ^a^VA administrative data assesses military sexual assault plus severe sexual harassment

Across the three missingness assumptions, the smallest discrepancy between combat estimates for men occurred among those told their name had been obtained from a list of OEF/OIF/OND Veterans. At 19 percentage points, however, the discrepancy was still substantial. The smallest discrepancy between military sexual assault estimates for men, at 14.2 percentage points, was seen among men randomized to receive the $40 post-paid incentive. For women, the smallest discrepancies between combat estimates and military sexual assault estimates both occurred among those told the survey would ask about combat (8.6 percentage points and 1.2 percentage points, respectively).

## Discussion

Our findings showed that modifying the cover letter’s content to say how survey recipients’ names had been obtained and offering a more generous post-paid incentive increased net survey response rates by as much as 10 percentage points. Our hypotheses related to non-response bias were only partially supported. As we expected, male combat Veterans were over-recruited from the group told that the survey asked about combat, and male sexual assault survivors were under-recruited from the group told that the survey asked about unwanted sexual attention. Differences in what Veterans were told about the survey’s content explained almost a third of the variance in men’s combat non-response bias and a quarter of their military sexual trauma non-response bias. However, neither of these findings were statistically significant. Women combat Veterans were over-recruited across all the study-arm manipulations. When told the survey asked about unwanted sexual attention, women with those exposures were over-recruited, but again, at a statistically non-significant level. Except for over-recruiting women with Charlson scores > 0 in the group told their name came from a list of Veterans filing for disability benefits, none of our hypotheses related to telling Veterans how their name had been obtained were supported. A higher post-paid incentive likewise had no impacts on improved representativeness of Veterans recruited into the study. Men’s self-reported combat and military sexual assault exposures were most consistent with a non-random pattern of missingness across all the study-arm manipulations. Particularly for military sexual assault, women’s estimates were considerably closer across the 3 assumptions of missingness, suggesting that their missingness may have, in fact, been random for this variable. Interestingly, the smallest discrepancy in women’s combat and military sexual assault estimates occurred in the group told that the survey asked about combat.

We had anticipated that combat Veterans and Veterans applying for PTSD disability benefits would be particularly interested in participating in research geared to those topics. Topic interest is generally considered a positive leverage that facilitates research participation. Whether it results in over-representation by interested participants is less straightforward [33]. In a clear example of topic interest leading to over-representation, Groves et al. [13] showed that members of a birdwatching association were almost twice as likely to participate in a survey about birding than they were a survey about mall design. Furthermore, members were more than twice as likely as non-members to participate in the birding survey. On the other hand, Groves et al. [13] also showed that patients with diabetes were no more likely to take part in a survey about diabetes than they were to take part in a survey about life quality. We showed elsewhere that male Gulf War I Veterans were more likely to report combat in a mailed survey if they were assigned to a low privacy, low incentive condition compared to Veterans assigned to greater privacy/higher incentive conditions [15]. These results suggested that male combat Veterans were particularly motivated to participate in a survey asking about combat exposures. The direction of effects in the present study is consistent with this interpretation, though, again, findings were statistically non-significant. Interestingly, even though women with combat exposures were over-represented in all our study-arms, their self-reported estimates of combat exposure were closer to a random missing pattern than were the men’s.

High-threat questions, such as asking about sexual victimization, are generally considered negative leverages that may discourage research participation, particularly by those who have experienced unwanted sexual attention. We previously showed that male Gulf War I Veterans who applied for PTSD disability benefits and had a history of military sexual trauma were particularly unlikely to participate in a survey asking about such experiences [8]. Their self-reported sexual assault experiences also showed a non-random pattern of missingness of a magnitude strikingly similar to what we report here. In contrast, telling women that the survey asked about unwanted sexual attention resulted in a slight, yet not statistically significant over-representation of those with military sexual trauma and the largest discrepancy between random and non-random missingness assumptions.

Although it increased net response rates, doubling our incentive had no statistically significant impacts on the bias in any of the 8 administrative variables in either gender, though it did explain almost 37% of the age bias in men and 19% of the bias in men’s Charlson Comorbidity scores. We had previously shown that a pre-paid $20 versus $10 incentive increased respondent representativeness by bringing younger and healthier participants to a survey about military traumas [15]. Possibly there are no further gains to be had by increasing incentives beyond $20. Other researchers have found mixed effects for the impact of incentives on bias [21, 23, 34]. Although incentives have been shown in several studies to enhance recruitment of African Americans [24], they had no impact on the under representation of nonwhites in the present study.

### Strengths and limitations

The study has several strengths, including its randomized, factorial design and its focus on an important, policy-relevant population. While a comprehensive literature examines the impact of various interventions on survey response rates, relatively few examine methods to reduce non-response bias. In terms of limitations, our response rate was lower than anticipated, particularly in some factorial combinations. Findings are therefore susceptible to Type II error. The study also does not answer how large non-response bias can be before it becomes intolerable. Our data indicate that, in some cases, non-response biases of less than 10 absolute percentage points in men were associated with over-estimations of combat exposure and under-estimations of military sexual assault of almost 30 percentage points. Distortions of this magnitude could lead to misallocating resources—for example, underfunding military sexual assault treatment for men. VA administrative data are not perfect indicators of Veterans’ true combat and military sexual trauma status and probably underestimate both. However, positive values tend to be correct and thus relate informatively to self-reported values. The study also targeted OEF/OIF/OND Veterans applying for PTSD disability benefits. This is a highly selected group, and the manipulations we applied to the cover letters were uniquely targeted to them. Results may not apply to other populations.

## Conclusions

Researchers, e.g., [13, 24], have always acknowledged that higher response rates could counterintuitively increase non-response bias were the higher response rate achieved by over recruiting subgroups with specific attributes. This study offers several examples of where this occurred. VA administrators need to be aware that trauma surveys likely overestimate combat and underestimate sexual assault in male PTSD disability applicants by a substantial degree. This information should be kept in mind when allocating scarce resources to address these issues. Specialized methods, which might include targeted recruitment methods for selected subgroups, such as men under the age of 30 or non-white women, must be used to ensure adequate representation of undercounted Veterans. Recently, Gray et al. [35] offered a range of population estimates for harmful drinking using several plausible missingness patterns, Reporting study results under different assumptions of missingness allows readers to concretely see the impact of these assumptions on study estimates.

## Supplementary Information

**Additional file 1: Table S1.** Men and Women’s Survey Response (n) and Response Rate (%) by each Factorial Combination. **Table S2a**. Mean bias (SD) by study-arm manipulation for men*.* **Table S2b**. Mean bias (SD) by study-arm manipulation for women.

## Data Availability

The data that support the findings of this study are available from the corresponding author (MM) upon reasonable request and with local Minneapolis VA Health Care System IRB permission.

## Abbreviations

ANOVA: Analysis of variance
ICD-9-CM: International Classification of Diseases, Clinical Modification, 9th revision
ICD-10-CM: International Classification of Diseases, Clinical Modification, 10th revision
MST: Military sexual trauma
OEF/OIF/OND: Operations Enduring Freedom, Iraqi Freedom, or New Dawn
PTSD: Posttraumatic stress disorder
SAS: Statistical Analysis System
SMI: Serious mental illness
SPSS: Statistical Package for the Social Sciences
SSI: Intervention Sum of Squares
SST: Total Sum of Squares
VA: Department of Veterans Affairs
