# Supplementary material for: Impact of different cover letter content and incentives on non-response bias in a sample of Veterans applying for Department of Veterans Affairs disability benefits: a randomized, 3X2X2 factorial trial
Source: BMC Med Res Methodol. 2022 Mar 6;22:61. doi: 10.1186/s12874-022-01531-x (PMC8898515; doi:10.1186/s12874-022-01531-x)
Supplement: Supplementary file 1 — Additional file 1: Table S1. Men and Women’s Survey Response (n) and Response Rate (%) by each Factorial Combination. Table S2a. Mean bias (SD) by study-arm manipulation for men. Table S2b. Mean bias (SD) by study-arm manipulation for women. [file 12874_2022_1531_MOESM1_ESM.docx]

**Supplementary Table 1.**

Men and Women’s Survey Response (n) and Response Rate (%) by each Factorial Combination

| Gender | Factor 2: How Name was Obtained | Factor 1: What Veterans were told about the survey’s content | | | | | |
| --- | --- | --- | --- | --- | --- | --- | --- |
|  |  | Survey Asks about Combat | | Survey asks about unwanted sexual attention | | Survey asks about lifetime and military experiences | |
|  |  | Factor 3: Honoraria | | Factor 3: Honoraria | | Factor 3: Honoraria | |
|  |  | $20 | $40 | $20 | $40 | $20 | $40 |
| Men | From list of OEF/OIF/OND Veterans | 13 (32.5%) | 21 (52.5%) | 16 (40.0%) | 15 (37.5%) | 9 (22.5%) | 14 (35.0%) |
|  | From a list of Veterans applying for disability benefits | 20 (50.0%) | 16 (40.0%) | 14 (35.0%) | 24 (60.0%) | 14 (35.0%) | 23 (57.5%) |
| Women | From list of OEF/OIF/OND Veterans | 15 (37.5%) | 16 (40.0%) | 16 (40.0%) | 24 (60.0%) | 13 (32.5%) | 18 (45.0%) |
|  | From a list of Veterans applying for disability benefits | 16 (40.0%) | 17 (42.5%) | 14 (35.0%) | 21 (52.5%) | 20 (50.0%) | 21 (52.5%) |

OEF/OIF/OND = Operation Enduring Freedom, Operation Iraqi Freedom, Operation New Dawn.

Omnibus χ^2^ test for differences across response rates for men = 6.84, degrees of freedom = 2, *p* = 0.03. Omnibus χ^2^ test for differences across response rates for women = 0.25, degrees of freedom = 2, *p* = 0.88.

**Supplementary Table 2a.**

Mean bias (SD) by study-arm manipulation for men*.* Results are reported as percentage points

| Characteristic as obtained from VA databases | Study-Arm Manipulation | | | | | | |
| --- | --- | --- | --- | --- | --- | --- | --- |
|  | What Veterans were told about content | | | How name was obtained | | Honoraria | |
|  | Combat | Unwanted Sexual Attention | Lifetime/  military experiences | List of OEF/OIF/  OND Veterans | List of Veterans Applying for disability benefits | $20 | $40 |
| Age < 30 | -4.54 (8.9) | -10.18 (7.68) | -5.10 (3.68) | -5.81 (4.61) | -7.4 (9.15) | -10.63 (5.75) | -2.58 (5.87) |
| Non-white race | 0.54 (8.78) | 4.33 (6.55) | -3.96 (5.75) | -0.30 (6.34) | 0.91 (8.84) | 1.07 (8.74) | -0.46 (6.44) |
| Combat exposure | 4.59 (10.34) | -0.37 (3.85) | -9.82 (13.90) | -1.90 (12.67) | -1.84 (10.69) | -6.42 (13.27) | 2.69 (6.99) |
| Military sexual trauma exposure | 1.17 (1.37) | -2.5 (0.00) | -0.12 (4.12) | -1.94 (1.13) | 1.47 (3.00) | 0.54 (3.61) | -1.00 (1.84) |
| Serious mental illness | 0.86 (6.30) | 0.37 (4.07) | 5.40 (9.41) | 4.15 (8.36) | 0.27 (4.50) | 0.91 (6.63) | 3.51 (7.16) |
| Charlson Comorbidity Index > 0 | 3.13 (5.32) | 2.96 (12.29) | -1.64 (5.60) | 4.65 (9.34) | -1.69 (5.18) | -1.84 (5.21) | 4.80 (9.20) |
| VA disability benefits for PTSD | 3.11 (6.45) | -2.16 (6.38) | 4.84 (14.08) | 2.34 (10.77) | 1.53 (8.56) | 4.32 (12.29) | -0.46 (4.99) |
| Any VA disability benefits | 4.41 (8.81) | 4.14 (8.47) | 8.64 (4.79) | 6.95 (8.11) | 4.51 (6.66) | 5.14 (7.24) | 6.32 (7.77) |

OEF/OIF/OND = Operation Enduring Freedom, Operation Iraqi Freedom, Operation New Dawn. VA = Department of Veterans Affairs. PTSD = Posttraumatic stress disorder. Positive numbers indicate over-representation among respondents and negative numbers indicate under representation.

**Supplementary Table 2b.**

Mean bias (SD) by study-arm manipulation for women*.* Results are reported as percentage points

| Characteristic as obtained from VA databases | Study-Arm Manipulation | | | | | | |
| --- | --- | --- | --- | --- | --- | --- | --- |
|  | What Veterans were told about content | | | How name was obtained | | Honoraria | |
|  | Combat | Unwanted Sexual Attention | Lifetime/  military experiences | List of OEF/OIF/  OND Veterans | List of Veterans Applying for disability benefits | $20 | $40 |
| Age < 30 | -1.82 (7.75) | -6.04 (8.52) | 2.05 (1.86) | -1.69 (8.85) | -2.18 (5.43) | -1.31 (8.73) | -2.57 (5.56) |
| Non-white race | **-6.55 (4.28)** | **-6.40 (3.32)** | **-0.25 (4.98)*** | **-7.15 (3.67)** | **-1.65 (4.4)*** | -4.33 (5.02) | -4.47 (5.28) |
| Combat exposure | 4.49 (7.61) | 3.50 (8.39) | 5.91 (6.62) | 0.97 (3.80) | 8.30 (7.68) | 5.17 (5.77) | 4.10 (8.47) |
| Military sexual trauma exposure | 1.16 (6.23) | 2.99 (5.82) | -7.74 (10.71) | -3.58 (10.37) | 1.19 (6.62) | -0.65 (8.70) | -1.74 (9.42) |
| Serious mental illness | 3.38 (1.22) | 7.09 (6.26) | -0.85 (6.23) | 3.60 (8.48) | 2.85 (3.85) | 2.92 (7.80) | 3.52 (3.25) |
| Charlson Comorbidity Index > 0 | 1.17 (5.49) | 3.26 (7.22) | -1.87 (4.79) | **-3.21 (2.86)** | **4.92 (5.09)*** | 2.01 (7.31) | -0.30 (4.13) |
| VA disability benefits for PTSD | 2.87 (3.85) | 1.61 (12.94) | 10.47 (10.42) | 1.30 (6.25) | 8.67 (11.81) | 7.74 (12.87) | 2.23 (5.23) |
| Any VA disability benefits | **9.33 (5.62)** | **-0.06 (3.90)** | **-3.61 (7.95)*** | -0.72 (7.83) | 4.49 (7.74) | 0.99 (11.08) | 2.78 (3.57) |

OEF/OIF/OND = Operation Enduring Freedom, Operation Iraqi Freedom, Operation New Dawn. VA = Department of Veterans Affairs. PTSD = Posttraumatic stress disorder. Positive numbers indicate over-representation among respondents and negative numbers indicate under representation. **Bold face font** signifies a statistically significant difference within study manipulation for that characteristic. **p* < 0.05
